# Normativity vs. uniqueness: effects of social relationship strength on neural representations of others

**DOI:** 10.1093/scan/nsae045

**Published:** 2024-06-25

**Authors:** Taylor D Guthrie, Robert S Chavez

**Affiliations:** Department of Psychology, University of Oregon, Eugene, OR 97403, United States; Department of Psychology, University of Oregon, Eugene, OR 97403, United States

**Keywords:** social cognition, person perception, social relationship, normative, individuation

## Abstract

Understanding others involves inferring traits and intentions, a process complicated by our reliance on stereotypes and generalized information when we lack personal information. Yet, as relationships are formed, we shift toward nuanced and individualized perceptions of others. This study addresses how relationship strength influences the creation of unique or normative representations of others in key regions known to be involved in social cognition. Employing a round-robin interpersonal perception paradigm (*N* = 111, 20 groups of five to six people), we used functional magnetic resonance imaging to examine whether the strength of social relationships modulated the degree to which multivoxel patterns of activity that represented a specific other were similar to a normative average of all others in the study. Behaviorally, stronger social relationships were associated with more normative trait endorsements. Neural findings reveal that closer relationships lead to more unique representations in the medial prefrontal cortex and anterior insula, areas associated with mentalizing and person perception. Conversely, more generalized representations emerge in posterior regions like the posterior cingulate cortex, indicating a complex interplay between individuated and generalized processing of social information in the brain. These findings suggest that cortical regions typically associated with social cognition may compute different kinds of information when representing the distinctiveness of others.

## Introduction

Social cognition is anchored heavily on our ability to infer the intentions, character, and preferences of another’s mind, despite being fundamentally blind to the true source of this information. Initially, in the absence of direct experience or prior interaction, our brains default to relying on broad, categorical assumptions to navigate potential interactions with new acquaintances (Chan et al. 2011, Biesanz 2021, Rogers 2021). Such early interactions are often overshadowed by stereotypes and generalizations, which effectively strip the individual of their unique identity. However, it is through deliberate and effortful engagement that a transformative process begins to unfold. This process gradually dismantles biases, allowing individuation and nuanced understanding to enrich the mental model we construct of that person (Haslam 2006, Swencionis and Fiske 2013). How, though, does this transition from generalized perceptions to nuanced individualized understanding manifest in the brain?

In this study, we test the hypothesis that the strength of social relationships directly influences how the brain represents others, leading to increasingly individualized perceptions within regions of the brain critical for person perception and social cognition more broadly. This hypothesis builds on the premise that familiarity and emotional closeness not only deepen our understanding of others (Fiske and Neuberg 1990) but may also modify the neural underpinnings of this perceptual process. Our research diverges from directly testing the out-group homogeneity effect (Tajfel 1970), aiming instead to test a novel but related hypothesis that gradients of individualization may exist within pre-established social networks. Specifically, we examine whether the strength of social ties within a group of acquaintances can predict the extent to which a neural representation of a specific group member deviates from an averaged “normative other” activity pattern.

To test the theory that social relationships influence the creation of more unique brain representations, we used a round-robin design. Investigating true relationships in cognitive neuroscience is crucial, as it provides deeper insights into the cognitive processes that unfold in real-world social interactions. Traditionally, research in person perception has often relied on the use of stimuli that describe fictional or socially distant individuals (Kelley et al. 2002, Hassabis et al. 2014, Tavares et al. 2015), leaving room for potential limitations in the ecological validity of the findings. Additionally, studies that have used subjects’ own close others as stimuli (Chavez et al. 2017, Thornton and Mitchell 2017) have encountered challenges in controlling for unique variables inherent in each relationship, such as the degree of closeness, similarity, and the history of interactions, which can vary widely across participants. The round-robin design offers a robust solution to these challenges by involving small groups of individuals who are already familiar with each other to varying extents (Chavez and Wagner 2020, Guthrie et al. 2022). This method allows researchers not only to probe the cognitive responses of perceivers in a more controlled and authentic context but also to demonstrate how these perceptions are influenced by the varying dynamics within the group relationships. As a result, this approach allows for an investigation of genuine interpersonal relationships, offering valuable insights into the cognitive processes underpinning person perception.

Influential behavioral theories on impression formation highlight our tendency to rely on generalized or normative information when understanding others (Cronbach 1955, Fiske and Neuberg 1990, Biesanz 2021, Rogers 2021). This approach is advantageous when meeting someone new, given that, statistically, a random person is much more likely to resemble the average person than any specific known other (Chan et al. 2011, Rogers 2021). While much of the research has focused on the mechanisms of impression formation, certain studies have explored how our dependence on normative profiles is affected by the depth of social relationships or the passage of time (Biesanz et al. 2007, Human et al. 2020). These studies, however, have led to conflicting results with some showing a decrease in the use of normative profiles over time or as relationships emerge and others showing an increase. Furthermore, it has been suggested that specific social goals encourage us to differentiate individuals (Fiske and Neuberg 1990), for reasons ranging from enhancing group identity to fostering trust (Fiske et al. 1999). Despite this rich landscape of behavioral insights, a gap remains in our understanding of how the brain balances normative and individuated representations in the context of others, particularly regarding how this may be influenced by the strength of social relationships.

Much of the research that has extended this social psychological work on person perception into the neural domain has focused on impression formation and the role of stereotypes in categorizing others (Quadflieg et al. 2011, Freeman et al. 2012, Stolier and Freeman 2016, Brooks and Freeman 2019, Barnett et al. 2021). This work, along with research on person perception and social cognition more broadly, has led to the discovery of a broad network of regions that collectively allow the brain to process perceptual features of a particular other while also allowing for the inclusion of social information in the perceptual process (Saxe and Kanwisher 2003, Wagner et al. 2012, Brooks and Freeman 2019). Notably, it has been demonstrated through the use of multivoxel pattern analysis that generalized information, such as gender or race, can be detected within this network (Contreras et al. 2013, Stolier and Freeman 2016, Brooks and Freeman 2019). However, additional studies suggest that stimulus identity can also be decoded within these socially oriented brain regions, indicating that the brain can also utilize a unique representational structure when forming perceptions of others (Axelrod et al. 2015). This dual aspect of brain function within this brain network underscores the social cognitive capacity to move from generalized to unique perceptions of others depending on the context of the situation.

Round-robin designs are uniquely positioned to advance our understanding of normative profiles in the brain. They enable the modeling of multiple representations of known others within each subject and permit the analysis of how various types of relationships might influence the differences in these representations (Guthrie et al. 2022). By utilizing the extensive data on known others represented in each subject’s brain throughout the study, this method enables the generation of normative brain patterns through the aggregation of these individual representations. This approach may provide a more accurate reflection of how generalized representations are used in real-world settings. Additionally, unlike studies that focus on perceiving a single known individual (Chavez et al. 2017), this design can differentiate whether subjects are using generalized or unique cognitive representations based on their relationship with each target, offering a more nuanced understanding of how these brain regions are processing social information.

To this end, the current study utilized functional magnetic resonance imaging (fMRI) to investigate the hypothesis that greater levels of social relationship strength would result in more distinct neural multivoxel patterns in socially oriented brain regions. By employing a round-robin interpersonal perception paradigm, we were able to collect behavioral and neuroimaging data from 20 pre-existing groups of five to six people from various real-world social networks. The study design allowed us to create an averaged “normative other” brain representation from brain responses each subject exhibited while thinking of each of their group members across the entire dataset. This normative pattern was then used to test whether social relationship metrics could predict the degree to which a subject was using either an average representation of a particular other or whether they had formed a more unique multivoxel pattern of activity to represent their group member. This approach, combined with the behavioral measures of social relationship strength, allowed us to examine whether gradients of individualization exist within pre-established groups and whether social relationships modulate the way that our brains model these perceptions.

## Materials and methods

### Participants

Using a multigroup round-robin design, we recruited a total of 120 right-handed participants between the ages of 18 and 51 years (48 females and 72 males) from 20 pre-existing independent social network groups, including student organizations, local businesses, and friend groups. Participants ranged in age from 18 to 51 years with a mean age of 23.5 years and a standard deviation of 7.2. A majority of participants identified as Caucasian (77.9%), 4.4% identified as Black or African American, 2.7% identified as Asian, 1.8% identified as American Indian or Alaska Native, 1.8% identified as Native Hawaiian or Pacific Islander, 9.7% identified as more than one race, and 1.8% chose not to identify their race. A majority of participants identified as non-Hispanic (84.1%). Six people were recruited from each of the 20 groups, and all participants within each group were familiar with one another but had various degrees of closeness with the other members.

### Procedures

#### Behavioral

Across all groups, five participants failed to fulfill scheduled experimental session appointments, two subjects were excluded due to unusable imaging data, and two subjects were excluded for missing or incomplete behavioral data. This left a final total of 111 subjects, with at least five participants per group. All participants were screened for magnetic resonance imaging (MRI) contraindications and had normal or corrected-to-normal vision. Each participant took part in two sessions. The initial session focused on behavioral ratings, during which participants completed a set of questionnaires for themselves and their fellow group members. The second session was an MRI scanning session, where participants made trait judgments for both themselves and their peers while undergoing functional neuroimaging. Participants provided informed consent in accordance with the guidelines established by the Internal Review Board at the University of Oregon for each session and received compensation for their involvement after completing each part of the study.

Participants were brought into the laboratory for the first session and tasked with responding to a series of inquiries about themselves and a specific group of known peers. Each participant was requested to provide ratings of closeness and similarity toward each individual in the group. All responses were logged using PsychoPy stimulus presentation software (Peirce et al. 2019). During the behavioral assessment, participants viewed a range of scales (ratings 1–5), with either one of their peers’ names or their own displayed above each scale. A single question was presented at the top of the screen, prompting participants to rate their peers or self-assess on that particular characteristic. In order to ensure comprehensive feedback, participants had to rate every peer before proceeding to subsequent questions. The entire session took ∼1 h.

The ratings gathered during this session were then utilized to compute the behavioral interpersonal relationship evaluations for subsequent fMRI studies. These assessments were derived from self-reported responses on a 1–5 Likert scale related to various statements about friendship, knowledge of the individual, affinity, and perceived similarity. These responses were combined into an overall measure of social relationship strength. This approach mirrored the one employed by Guthrie et al. (2022) and was reassessed for its application in the normative analyses.

#### Neuroimaging

In a separate laboratory session, the participants returned to complete the fMRI part of the study. While in the scanner, they were tasked with a common trait judgment activity that has been widely used in research on self and other processing (Kelley et al. 2002, Mitchell et al. 2011). The study employed a comprehensive round-robin design, meaning each participant acted as both an evaluator and a target stimulus for every other participant. A screen displayed two words arranged vertically in white text on a black background. For each trial, the top word showed either “Self” or one of five group members’ names from their own group. During an initial session outside of the scanner, participants indicated which name they most frequently used when referring to each group member. These names were then utilized during fMRI sessions (e.g. “Alexander” might have been changed to “Alex” or “Zander”). This was done to ensure that participants did not need extra time inside the scanner to try to figure out who each target was.

The bottom word presented one of 60 valence-balanced adjectives (e.g. “Happy,” “Clumsy,” and “Smart”; Anderson 1968) for 2000 ms followed by fixations lasting 2000–12 000 ms interspersed with intermittent passive fixation trials. Jittered trials were optimized using Opseq2 (Dale 1999). Participants had to indicate whether a given trait adjective described themselves or one of their group members by pressing buttons labeled “yes” or “no.” All targets appeared in each run with a total of 12 trials per target per run where all targets were paired with these same 12 trait adjectives. The same targets were then used in each subsequent run but were then paired with a new set of 12 trait adjectives in each one, resulting in all targets being paired with all 60 trait adjectives over the course of the experiment. Individual traits were only presented once per target randomly across all runs in the experiment. No two participants were presented with the same target/trait adjective order across the experiment to account for potential order effects.

MRI was conducted with a Siemens 3T Skyra scanner using a 32-channel phased array coil. Structural images were acquired using a T1-weighted MP-RAGE protocol [175 sagittal slices; time repetition (TR): 2500 ms; time echo (TE): 3.43 ms; flip angle: 7°; 1 mm isotropic voxels]. Functional images were acquired using a T2*-weighted echo planar sequence (TR: 2000 ms; 72 axial slices; TE: 25 ms; flip angle: 90°; 2 mm isotropic voxels). For each participant, we collected five runs of the round-robin task (188 whole-brain volumes per run). In order to correct for distortion due to B0 inhomogeneity, we also acquired a field map (TR: 6390 ms; TE: 47.8 ms; effective echo spacing: 0.345 ms). The total length of time for the entire scanning session was ∼75 min, and each of the five functional runs was ∼6 min long.

### Analysis

#### Preprocessing

Functional imaging data acquired for the fMRI task underwent preprocessing and the estimation of voxel responses was completed using the FMRIB Software Library (FSL) (Smith et al. 2004). The data went through mean-based intensity normalization, high-pass filtering (Gaussian-weighted least-squares straight line fitting with sigma = 100 s), and spatial smoothing with a 4-mm full width at half maximum Gaussian smoothing kernel. A multistep normalization procedure was employed to register the results to standard space. First, functional data were corrected for spatial distortion by using a field map unwarping before aligning functional data to each participant’s anatomical scan by using boundary-based registration (Greve and Fischl 2009) in conjunction with a linear registration with FSL’s FLIRT tool. These images were then warped to a 2-mm Montreal Neurological Institute (MNI) template by using nonlinear registration with FSL’s FNIRT tool and a 10-mm warp field. All first-level task-specific analyses were initially conducted in native space before being warped into standard space for final analyses. Parameter estimates were independently calculated for each of the five group members within all five runs completed by each participant. These responses were then combined in a second-level within-subject fixed-effects analysis yielding parameter estimates for each of the five group members’ “target” conditions. Although a self-condition was collected during this experiment, none of the self-estimates were used in this particular analysis. All estimates used in the analysis represented perceivers thinking of other group members. Normalized (i.e. *z*-score) voxel responses for each condition were extracted from a set of 400 parcels from the Schaefer atlas (Schaefer et al. 2018).

#### Normative other estimation

**Figure 1.**
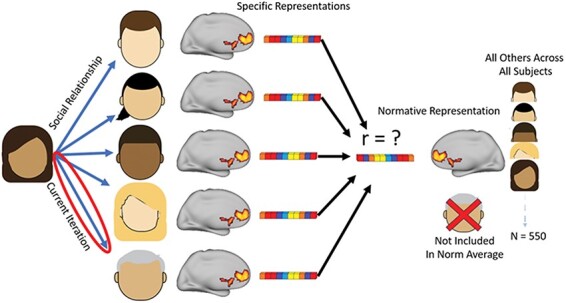
fMRI analysis was performed by acquiring brain activity estimates for each group member, segmenting them into 400 regions, creating a normative average pattern for each region excluding the specific representation being compared, and calculating Spearman’s rank correlation distance between the specific representation and normative average to measure dissimilarity, which was then predicted by the strength of the social relationship between the subject and each group member.

The result of the response pattern estimation process was a set of five estimates (one for each perceived target) in each of the 400 parcellated regions of interest (ROIs) for every one of the 111 subjects. Across all subjects included in the analysis, there were 555 unique estimates of cognition linked to a perceiver thinking about a known other. Parameter estimates within each ROI were flattened into 1D response vectors to allow for the alignment of responses across subjects. Similar to the procedure used when calculating a normative personality profile (Rogers 2021), the 1D response vectors for all unique estimates in each ROI across all of the subjects were averaged to allow for the calculation of a normative other estimate. This estimate then became the anchor of comparison in each ROI for calculating the dissimilarity between a specific representation of a particular other and the normative averaged response of all others in the study. It is important to note that the particular target that was being compared to the normative average was systematically left out of the calculation of the normative average in each iteration of the analysis to avoid a potential biasing of the correlation distance between that target’s particular representation and the norm. This meant that none of the estimates of the perceiver thinking about that target were included in the average, nor were any of the estimates created by other group members thinking of that same target. A schematic of this approach is shown in Fig. 1.

#### Distance between specific others and normative average

Consistent with previous studies examining the similarity/dissimilarity of neural representations (Kriegeskorte 2008), we computed, within each subject, the dissimilarity between neural response patterns of specific peer response vectors from the averaged normative other response vectors by using Spearman’s rank correlation distance. Within each parcel, we calculated correlation distances between a subject’s representation of a particular other and the normative other estimate, and this was done iteratively for all five group members that the subject perceived in the experiment. These correlation distances were then related to social relationship scores that were obtained in the behavioral session in which each perceiver rated the other members in their group. We sought to determine whether the distance in multivoxel similarity between each particular other and the normative average was related to social relationship strength. In order to account for the nested group structure of the comparisons, a linear mixed-effects model was employed in which the perceiver was modeled as a random intercept nested within each group. False discovery rate (FDR) correction was applied to correct for multiple comparisons across the 400 parcel ROIs.

#### Trait endorsement analysis

In addition to the neuroimaging data, we also analyzed the trait endorsement ratings collected during the scanner session to investigate whether the strength of social relationships influenced the similarity between an individual’s trait endorsements for a specific target and the normative endorsement pattern derived from all other subjects rating other targets. By comparing the results of this endorsement analysis with the findings from the neuroimaging data, we aimed to gain a more comprehensive understanding of how social relationships shape both explicit behavioral responses and implicit neural representations in the context of person perception. For each subject, we extracted the endorsement ratings, indicating whether they responded “yes” (the trait described the target) or “no” (the trait did not describe the target) to the trait adjectives paired with each target in each run.

To perform an analysis like the one conducted on the brain data, we first sorted the dataset in alphabetical order by trait word to ensure that endorsement ratings were matched across targets and subjects. For each target, we isolated their endorsement ratings and calculated a normative endorsement by averaging the endorsement ratings for all other subjects rating all other targets, excluding the specific target being analyzed. We then computed the Euclidean distance between the target’s endorsement vector and the normative endorsement vector. This process was repeated for all possible combinations of targets and subjects across the dataset.

To investigate the relationship between social relationship strength and the similarity of endorsement ratings to the normative pattern, we extracted the social relationship scores for each subject rating the particular target used in the analysis. These scores were then used as predictors in a linear mixed-effects model, with the Euclidean distance values as the dependent variable. The model included subjects nested within groups as a random effect to account for the hierarchical structure of the data.

## Results

**Figure 2.**
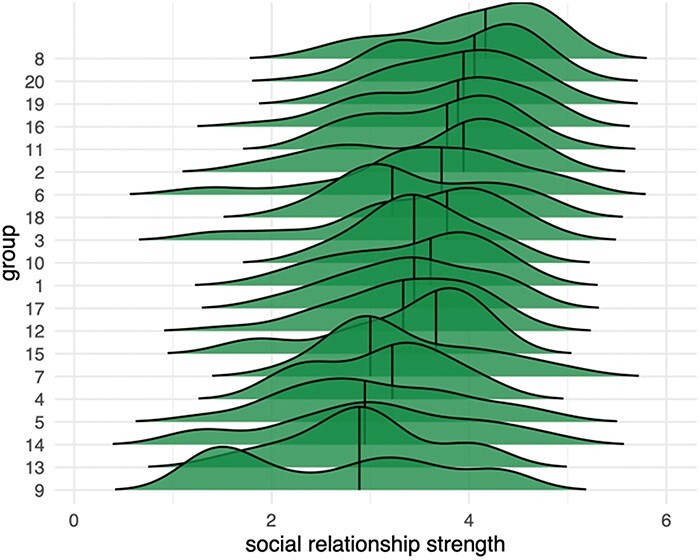
Variability in social relationship strength across 20 different groups. The ridgeline plot illustrates the distribution of perceived relationship strength within each group, highlighting both within-group variability and between-group differences. Groups exhibit a range of relationship strengths, with some groups reporting stronger ties and others weaker connections. Groups were deliberately recruited to enhance variability, including friend groups, academic peers, and work acquaintances, to capture a wide spectrum of familiarity.

Participants were recruited from real-world social groups in which each person had previously established relationships with all other members within each round-robin group. Despite the previous acquaintanceship, there remained a high degree of social relationship strength variability across all participants (*M* = 3.46, SD = 0.83). Moreover, variability in social relationship strength was observed both within groups and between groups, with some groups having higher average social relationship strength than others. Means and distributions of social relationship strength for each group are shown in the density ridgeline plots in Fig. 2. Thus, as expected, these groups of previously acquainted individuals tended to have higher social relationship strength than would be expected of strangers, but the variability needed to model the fMRI similarity metrics was present in all groups.

**Figure 3.**
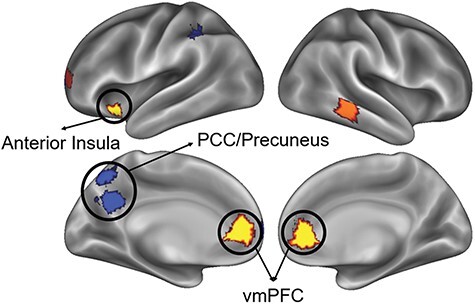
Brain regions exhibiting significant relationships with social relationship strength, where warm colors indicate areas in which stronger social ties predict greater uniqueness in neural representations of group members compared to the normative average, while cool colors mark regions where stronger social relationships correspond to more generalized neural patterns aligning with the normative group average.

All fMRI analyses were performed in the same manner within each of the 400 parcels independently, and significance values were corrected for multiple comparisons using FDR. We hypothesized that greater social relationship strength would be reflected in more unique (i.e. lower degree of similarity) multivoxel pattern similarity between an individual target and the normative other. The results of these analyses are displayed in Fig. 3. Consistent with this hypothesis, social relationship strength between a perceiver and a target was significantly associated with a greater degree of representational uniqueness within brain regions implicated in social cognition. These regions include the left ventromedial prefrontal cortex (vmPFC) [*b*  = 0.006 , standard error (SE) = 0.004, *P* < .001], the right vmPFC (*b*  = 0.005, SE = 0.001, *P* = .003), and the left ventral anterior insula (*b* = 0.006, SE = 0.001, *P* = .010). A complete list of all significant regions from these analyses is summarized in Table 1.

**Table 1.** Brain regions with unique multivoxel patterns predicted by social relationship strength.

Brain region	Peak coordinates (*x*, *y*, *z*; MNI)	Estimate	Standard error	*P*-value (FDR-corrected)
Left vmPFC	(−6, 45, 9)	0.006	0.004	<.001
Left ventral anterior insula	(−34, 16, −9)	0.006	0.001	.010
Left dorsolateral prefrontal cortex	(−27, 55, 12)	0.003	0.0009	.050
Right vmPFC	(7, 42, 5)	0.005	0.001	.003
Right middle temporal sulcus	(62, −41, −9)	0.003	0.0009	.021

Notes: This table lists brain regions where the strength of social relationships was found to significantly predict the uniqueness of multivoxel pattern activity, as indicated by greater dissimilarity from the normative average. Peak coordinates are presented in MNI space. *P*-values are FDR-corrected for multiple comparisons.

Although our hypothesis predicted that stronger social ties would be associated with greater uniqueness in representations of specific others, some regions showed an inverse relationship. In these areas, higher social relationship strength was associated with representations of specific others that were more closely aligned with the normative average (higher degree of similarity). The observations in the left posterior cingulate cortex (PCC)/precuneus region fall within an area known to be involved in social cognition. This included a more ventral region that overlaps with the PCC and precuneus (*b*  = −0.003 , SE = 0.0009, *P* = .012) and a more dorsal region within the precuneus (*b*  = −0.003 , SE = 0.0009, *P* = .039). These results are also shown in Fig. 3, and a complete list of all significant cluster statistics for the individual target results can be found in Table 2.

**Table 2.** Brain regions with generalized multivoxel patterns predicted by social relationship strength.

Brain region	Peak coordinates (*x*, *y*, *z*; MNI)	Estimate	SE	*P*-value (FDR-corrected)
Left PCC/precuneus	(−5, −61, 30)	−0.003	0.0009	.012
Left precuneus	(−5, −65, 52)	−0.003	0.0009	.039
Left dorsolateral prefrontal cortex	(−20, 61, 8)	−0.003	0.0009	.050
Left intraparietal sulcus	(−44, −42, 47)	−0.002	0.0007	.039

Notes: This table lists brain regions where the strength of social relationships significantly predicted the generalization of multivoxel pattern activity, as indicated by greater similarity to the normative average. Peak coordinates are presented in MNI space. *P*-values are FDR-corrected for multiple comparisons.

### Trait endorsement results

To investigate the relationship between social relationship strength and the similarity of trait endorsements to the normative pattern, we conducted a linear mixed-effects analysis. The model included social relationship scores as predictors of the Euclidean distance between a target’s endorsement vector and the normative endorsement vector, with run nested within subject, further nested within dyad, as random effects.

The analysis revealed a significant effect of social relationship strength on the similarity of trait endorsements to the normative pattern (*β* = −0.03, SE = 0.004, *t* = −6.83, *P* < .001). Contrary to our expectations, the negative coefficient indicates that as social relationship strength increased, the Euclidean distance between a target’s endorsement vector and the normative endorsement vector decreased. In other words, stronger social relationships were associated with more normative or average trait endorsements.

## Discussion

Navigating the diverse connections of our daily social experience necessitates the flexible adaptation of perceptual strategies to effectively model our representations of others in accordance with the nature of our relationship with them. Generalized cognitive representations may serve as efficient heuristics for forming impressions of acquaintances or strangers (Chan et al. 2011, Rogers 2021), but with close others, it becomes increasingly important to capture their nuanced individuality in the mental models we form of them (Swencionis and Fiske 2013). Using a round-robin fMRI design, the results from this study demonstrated that social bonds have a modulatory effect on the ways in which neural representations of close others are formed. By establishing a “normative other” activity pattern, aggregated from the collective representations of others across the study, and contrasting individual representations against this norm, we show that the strength of social relationships predicts the uniqueness of neural representations of others in key regions involved in social cognition.

The vmPFC plays a pivotal role in the process of person perception, as it has been shown to be preferentially engaged in the high-level abstract judgments required for making attributions about enduring traits (Mitchell et al. 2004, Harris et al. 2005, Schiller et al. 2009, Van Overwalle 2009, Ma et al. 2014a). Neuroimaging and virtual lesion studies have consistently shown that the vmPFC appears to be necessary and is also distinctly recruited when making these types of trait judgments, irrespective of general valence or the interpretation of specific behaviors in social stimuli (Van Overwalle 2009, Ma et al. 2014a, 2014b, Kestemont et al. 2016). This specificity suggests that trait information is not processed in a distributed manner across the networks involved in social cognition but rather that the vmPFC is a central component in the brain’s ability to create these types of representations. Furthermore, it has been demonstrated that the univariate activity patterns in the vmPFC increase linearly with the similarity and closeness of a social target being judged, highlighting that this trait space is likely utilized more with close or familiar others (Mitchell et al. 2006, Krienen et al. 2010).

Our study further elucidates the role of the vmPFC, revealing that it not only develops distinct neural activity patterns to represent others but also that these representations become increasingly more unique as the social relationship with the target increases. The distinct role of the vmPFC in creating personalized trait representations is crucial for the interpretation of these findings as the analyses were performed in dissimilarity space. Understanding that the vmPFC is likely engaged in computing high-level trait information allows us to infer that the normative pattern we have created represents an average trait space rather than an aggregate of neural responses to diverse cognitive processes such as attention or sensory processing. This suggests that the activity patterns observed in the vmPFC for specific targets diverge from this normative trait space and likely represent the nuanced ways in which we represent the specific traits of close others.

Another crucial component of creating and maintaining close relationships is the involvement of emotion. It has been demonstrated that along with the high-level trait attributions that the vmPFC is involved in, it has also been shown to represent affective meaning (Rudebeck et al. 2008, Roy et al. 2012). This is separate from general valence in that it requires an integration of valence signals with higher-level conceptual and contextual elements. Patients with lesions in the vmPFC have been shown to routinely exhibit irregular physiological and appraisal behavior in response to personally relevant social and emotional information (Damasio et al. 1990, Beer et al. 2003, Hornak et al. 2003, Leopold et al. 2012). Parallel to the findings in the vmPFC, we also found evidence for the unique representational structure effect in the anterior insula. This region is also heavily involved in the integration of emotion with higher-level subjective and social information (Kurth et al. 2010, Craig 2011, Chang et al. 2013, Yoon et al. 2021). Previous computational work also demonstrated the role of the anterior insula in social decision-making related to the creation of social alliances (Lau et al. 2020). Together, these findings demonstrate a possible individuation of trait information and affective meaning in these anterior social regions as social ties become more prominent with the individual that is being assessed.

It is widely recognized that the brain implements different learning strategies based on the context and demands of the situation (Schapiro et al. 2017). It is highly efficient for the brain to implement the use of prototypical heuristics to navigate scenarios that involve generalizable features, and the creation of these mental models likely involves the extraction of normative and consistent information that is routinely encountered (Bowman et al. 2020). To successfully navigate novel situations, however, it is also important to differentiate salient information that may be useful above and beyond the predictive power that a generalized prototypical model can afford. It is important to note, however, that the brain has been shown to be capable of creating and maintaining both types of representational structures simultaneously and in parallel (Schapiro et al. 2017). Discriminative attention has been proposed as a key factor in shifting the reliance away from a generalized representation by alternatively initiating a process that accommodates new information into the existing models (Kahneman et al. 1983, Musslick et al. 2020). Forming and maintaining strong, meaningful social bonds likely involves applying these discriminative attentional processes to recognize and appreciate the unique qualities that distinguish individuals from the broader collective (Fiske et al. 1999).

While we observed the anticipated unique representation effect in certain anterior areas within this network, such as the vmPFC and anterior insula, our findings also revealed an inverse effect in other regions known to be involved in social cognition. Notably, posterior regions, including parts of the PCC and precuneus, displayed patterns where stronger social ties resulted in a representational structure that was more generalized, converging more with the normative average. This divergence in neural processing between anterior and posterior regions suggests that the social networks in the brain do not operate uniformly. Instead, these anterior and posterior regions, which are often shown to be coactive in univariate studies (Raichle et al. 2001, Andrews-Hanna et al. 2014, Mak et al. 2017, Yeshurun et al. 2021), are likely engaging in separate and distinct computations when representing others. This highlights the role of the complementary learning systems approach and may be evidence for both generalized and individualized representations being maintained and utilized in parallel by different components of the network.

Although there was evidence of other regions showing the same pattern of unique brain activity being predicted by social relationship strength and other regions showing the inverse, these regions were outside of the hypothesized scope of the study and should be interpreted with caution. The analysis does indeed show that the patterns of activity in these regions are deviating from or converging with an average when thinking about close others, but it is unclear what that average or what the resulting idiosyncratic or generalized activity is computing. The brain is highly entangled, and it is likely that a shift in representational structure in the anterior social regions that we observed has a driving effect on other regions that may be close by or functionally coupled with these regions in ways that we have yet to understand (Chavez 2021). Additionally, these findings may suggest broader network changes in the way the brain orients itself to self-relevant information in general as close others are more likely to activate regions involved in the allocation of discriminative attention (Humphreys and Sui 2016) and the instantiation of long-term and working memory (Northoff et al. 2006, Wagner et al. 2012, Sui and Humphreys 2015). Although these unexpected findings offer intriguing insights into the complexity of neural processes, further research is needed for a full understanding of the significance and mechanisms underlying these phenomena. By investigating these regions further, future studies can shed light on the broader network dynamics associated with processing socially relevant information and elucidate their functional implications.

The unexpected finding that stronger social relationships were associated with more normative trait endorsements is intriguing and may be related to the results observed in the PCC and precuneus. The consistency between the endorsement findings and the PCC results suggests that there may be a common underlying mechanism driving both the behavioral and neural patterns. The PCC has been implicated in various aspects of social cognition, including self-referential processing, mentalizing, and integrating social information (Schilbach et al. 2008). It is possible that the PCC plays a role in aligning one’s perceptions and behavioral responses with social norms and expectations, particularly in the context of close relationships. The normative patterns observed in both the endorsement data and the PCC neural representations may reflect this social alignment process. Furthermore, this speaks to the conflicting results that have been observed in behavioral studies investigating normative trait profiles, with some of these investigations showing more normative trait assignment over time and others showing less (Biesanz et al. 2007, Human et al. 2020). These findings highlight the interplay between explicit behavioral responses and implicit neural representations in social cognition. Future studies should investigate the relationship between these different levels of analysis, particularly focusing on the role of the PCC in integrating social information and shaping both behavioral and neural patterns in the context of close relationships.

While this study provides evidence for the influence of social relationship strength on normative social cognition, it is also important to acknowledge a limitation in our design. All participants within each group possessed some level of prior acquaintance with all group members. As a result, there were no real strangers in our paradigm, which limits our ability to compare our findings to those of representations seen in the impression formation literature (Schiller et al. 2009, Brooks and Freeman 2019). Nevertheless, our findings demonstrate that despite this lack of complete unfamiliarity, the distinctiveness of representations still corresponded with the overall strength of social relationships. We demonstrated that there is indeed significant variability observed in the overall strength of relationships between perceivers and targets. By intentionally recruiting diverse groups, including individuals from different contexts such as friend groups, student groups, and work groups, we aimed to capture a range of relationship strengths. While this variability provides a more ecologically valid representation of real-world relationships, it can also complicate the interpretation of findings and may limit the generalizability of our results to specific relationship types. It is crucial to consider these limitations when interpreting the implications of our study, and future research should aim to address these issues for a more comprehensive understanding of the brain’s representation of close others.

## Conclusion

In summary, our research highlights a nuanced distinction in the neural mechanisms underlying social cognition, particularly the divergent roles of anterior and posterior regions in processing close others. Anterior regions, such as the vmPFC and ventral anterior insula, are shown to create unique, individualized representations of close others, emphasizing the brain’s capacity for integrating affective and cognitive information to support deep personal connections. Conversely, posterior regions, including the PCC and precuneus, demonstrate a tendency toward more generalized processing with stronger social ties, suggesting a complementary but distinct cognitive function from the anterior regions. Interestingly, our behavioral findings reveal that stronger social relationships are associated with more normative trait endorsements, which appears to align with the neural patterns observed in the posterior regions. This divergence between the behavioral and anterior neural results underscores the inherent interplay between explicit responses and implicit neural representations in social cognition. The distinction between anterior and posterior brain regions challenges previous assumptions that these areas operate uniformly to support social cognition. Instead, our findings propose that anterior and posterior brain regions engage in different types of cognitive processing when representing close others, indicating a more intricate and differentiated neural basis for person perception and social cognition more broadly. This work may inform future studies on the neural basis of strategies employed as social relationships evolve from zero acquaintance to intimate bonds, and it highlights the importance of considering both behavioral and neural measures to gain a comprehensive understanding of social cognitive processes.

## Data Availability

Data, materials, and codes used to generate the findings in this study are openly available via an Open Science Framework repository at https://osf.io/wnyma.
